# Effect of compensatory evolution in the emergence and transmission of rifampicin-resistant *Mycobacterium tuberculosis* in Cape Town, South Africa: a genomic epidemiology study

**DOI:** 10.1016/S2666-5247(23)00110-6

**Published:** 2023-07

**Authors:** Galo A Goig, Fabrizio Menardo, Zubeida Salaam-Dreyer, Anzaan Dippenaar, Elizabeth M Streicher, Johnny Daniels, Anja Reuter, Sonia Borrell, Miriam Reinhard, Anna Doetsch, Christian Beisel, Robin M Warren, Helen Cox, Sebastien Gagneux

**Affiliations:** a Medical Parasitology and Infection Biology, Swiss Tropical and Public Health Institute, Allschwil, Switzerland; b University of Basel, Basel, Switzerland; c Department of Plant and Microbial Biology, University of Zürich, Zürich, Switzerland; d Division of Medical Microbiology, Department of Pathology, University of Cape Town, Cape Town, South Africa; e Institute of Infectious Disease and Molecular Medicine and Wellcome Centre for Infectious Disease Research, University of Cape Town, Cape Town, South Africa; f Tuberculosis Omics Research Consortium, Family Medicine and Population Health, Institute of Global Health, Faculty of Medicine and Health Sciences, University of Antwerp, Antwerp, Belgium; g Department of Science and Innovation – National Research Foundation Centre of Excellence for Biomedical Tuberculosis Research, Stellenbosch University, Stellenbosch, South Africa; h South African Medical Research Council Centre for Tuberculosis Research, Division of Molecular Biology and Human Genetics, Faculty of Medicine and Health Sciences, Stellenbosch University, Stellenbosch, South Africa; i Médecins Sans Frontières, Khayelitsha, Cape Town, South Africa; j Department of Biosystems Science and Engineering, Eidgenössische Technische Hochschule Zürich, Zürich, Swizterland

## Abstract

**Background:**

Experimental data show that drug-resistance-conferring mutations are often associated with a decrease in the replicative fitness of bacteria in vitro, and that this fitness cost can be mitigated by compensatory mutations; however, the role of compensatory evolution in clinical settings is less clear. We assessed whether compensatory evolution was associated with increased transmission of rifampicin-resistant tuberculosis in Khayelitsha, Cape Town, South Africa.

**Methods:**

We did a genomic epidemiological study by analysing available *M tuberculosis* isolates and their associated clinical data from individuals routinely diagnosed with rifampicin-resistant tuberculosis in primary care and hospitals in Khayelitsha, Cape Town, South Africa. Isolates were collected as part of a previous study. All individuals diagnosed with rifampicin-resistant tuberculosis and with linked biobanked specimens were included in this study. We applied whole-genome sequencing, Bayesian reconstruction of transmission trees, and phylogenetic multivariable regression analysis to identify individual and bacterial factors associated with the transmission of rifampicin-resistant *M tuberculosis* strains.

**Findings:**

Between Jan 1, 2008, and Dec 31, 2017, 2161 individuals were diagnosed with multidrug-resistant or rifampicin-resistant tuberculosis in Khayelitsha, Cape Town, South Africa. Whole-genome sequences were available for 1168 (54%) unique individual *M tuberculosis* isolates. Compensatory evolution was associated with smear-positive pulmonary disease (adjusted odds ratio 1·49, 95% CI 1·08–2·06) and a higher number of drug-resistance-conferring mutations (incidence rate ratio 1·38, 95% CI 1·28–1·48). Compensatory evolution was also associated with increased transmission of rifampicin-resistant disease between individuals (adjusted odds ratio 1·55; 95% CI 1·13–2·12), independent of other patient and bacterial factors.

**Interpretation:**

Our findings suggest that compensatory evolution enhances the in vivo fitness of drug-resistant *M tuberculosis* genotypes, both within and between patients, and that the in vitro replicative fitness of rifampicin-resistant *M tuberculosis* measured in the laboratory correlates with the bacterial fitness measured in clinical settings. These results emphasise the importance of enhancing surveillance and monitoring efforts to prevent the emergence of highly transmissible clones capable of rapidly accumulating new drug resistance mutations. This concern becomes especially crucial at present, because treatment regimens incorporating novel drugs are being implemented.

**Funding:**

Funding for this study was provided by a Swiss and South Africa joint research award (grant numbers 310030_188888, CRSII5_177163, and IZLSZ3_170834), the European Research Council (grant number 883582), and a Wellcome Trust fellowship (to HC; reference number 099818/Z/12/Z). ZS-D was funded through a PhD scholarship from the South African National Research Foundation and RMW was funded through the South African Medical Research Council.

## Introduction

The emergence and transmission of drug-resistant *Mycobacterium tuberculosis* is threatening global tuberculosis control. Drug-resistant tuberculosis can emerge de novo through the selection of drug-resistant bacteria during patient treatment, or can reflect the patient-to-patient transmission of drug-resistant strains. In high-incidence settings, transmission, as opposed to de novo evolution, has been suggested to be the main cause of incident drug-resistant tuberculosis.^1^ Control of the drug-resistant tuberculosis epidemic requires effective strategies to stop transmission, particularly in settings where tuberculosis and HIV are endemic, where high rates of resistance because of transmission have been described.^2^ Mathematical models have identified the relative transmission fitness of drug-resistant bacteria compared with their drug-susceptible counterparts as a key factor in identifying the emergence and spread of drug-resistant tuberculosis.^3^, ^4^ However, current understanding of the factors influencing the transmission of drug-resistant tuberculosis between hosts is still low. Transmission of drug-resistant tuberculosis is a complex occurrence, established by many host and environmental variables. Among these, poverty, malnutrition, and the quality of tuberculosis control programmes have strong effects on the prevalence of tuberculosis in general, and drug-resistant tuberculosis in particular.^2^ By contrast, the influence of bacterial factors on drug-resistant tuberculosis transmission is less clear.

**Research in context**

**Evidence before this study**

Mutations conferring drug resistance in bacteria often cause a fitness defect, measured in terms of a reduced replicative capacity in culture. The acquisition of additional compensatory mutations can restore the replicative capacity of drug-resistant bacteria in vitro. In *Mycobacterium tuberculosis*, the fitness cost in vitro of different mutations conferring rifampicin resistance correlates with their relative clinical prevalence, suggesting an effect in the transmission of the disease. By contrast, the role of compensatory evolution in the transmission of rifampicin-resistant tuberculosis is controversial. We searched PubMed for published studies in English from database inception to Oct 24, 2022, with the keywords “tuberculosis”, “transmission”, and “compensatory”. Although five studies have suggested that rifampicin-resistant strains carrying compensatory mutations are more transmissible, others found no such relationship. However, previous studies have been limited to the use of genetic clustering or similar approaches as a surrogate of transmissibility. Moreover, previous studies focused on the role of bacterial genetic factors, independently from host-related factors.

**Added value of this study**

We used a Bayesian reconstruction of transmission trees to delineate the transmission of rifampicin-resistant tuberculosis in Khayelitsha, Cape Town, South Africa over a period of 10 years. We analysed the *M tuberculosis* genomes of individuals who were infected, along with the associated clinical data, to identify bacterial and host-related factors that increased the risk of rifampicin-resistant tuberculosis transmission. We identified several host and bacterial factors, including compensatory evolution, that were associated with an increased risk of drug resistance amplification and transmission.

**Implications of all the available evidence**

*M tuberculosis* strains carrying drug-resistance-conferring mutations, causing a low fitness cost and compensatory mutations, are more transmissible than those carrying mutations with a high fitness cost and without compensatory mutations in the high-incidence setting of Khayelitsha, South Africa. One question that has yet to be answered is to what extent the relationship between the different genetic determinants of resistance in *M tuberculosis* and its transmission depends on the epidemiological setting. Future research in different settings using methods capable of delineating transmission events is warranted.

Experimental studies in various bacterial species, including *Mycobacterium tuberculosis*, have shown that mutations conferring antibiotic resistance often confer a fitness cost in the absence of drugs, defined as a deficit in bacterial growth (ie, the replicative fitness).^5^ However, the magnitude of this cost in replicative fitness depends on the specific drug-resistance-conferring mutation, as well as on the epistatic interaction of this mutation with the bacterial genetic background. For example, in *M tuberculosis*, several studies have shown that the mutation *rpoB* S450L, which causes resistance to rifampicin, is associated with a smaller loss in replicative fitness compared with other *rpoB* mutations.^5^ This mutation is also the most common among rifampicin-resistant tuberculosis strains isolated from patients, suggesting that it is selected for in epidemiological settings.^6^ However, direct evidence supporting a higher transmission potential by strains carrying *rpoB* S450L among patient populations are few.

The role of compensatory mutations in alleviating the fitness cost of resistance has also been experimentally established in mycobacteria and other bacteria. In particular, compensatory mutations in *rpoA*, *rpoB*, and *rpoC* have been identified in rifampicin-resistant strains of multiple bacterial species, including *M tuberculosis.*^7^, ^8^ However, whether compensatory evolution also influences the transmission fitness of drug-resistant bacteria in epidemiological settings is a matter of debate. In *M tuberculosis*, some studies have reported an increased transmission fitness of drug-resistant strains carrying compensatory mutations,^9^, ^10^, ^11^, ^12^, ^13^ whereas others have found no effect.^14^, ^15^, ^16^, ^17^ There are different reasons that might explain these contradictory findings. One potential explanation is that the effect of compensatory evolution on transmission might vary across settings.^16^ Additionally, the term fitness has been used interchangeably to refer to different variables. In experimental work, fitness usually refers to the capacity of the bacteria to replicate in vitro. However, whether fitness, as measured in vitro, also increases the competitive fitness both within a host and during transmission between hosts, has not been explored. So far, the main limitation when evaluating the role of compensatory evolution in the transmission of drug-resistant bacteria has been the use of different measures of genetic distance (eg, clustering or terminal branch lengths in a phylogeny) as a proxy for transmissibility. However, there is increasing evidence that this approach is subject to several limitations that can confound the analysis.^18^ Another relevant limitation in previous studies is that the relationship between the genetic determinants of resistance in *M tuberculosis* and its transmission is often analysed independently from patient-related factors, some of which can be linked to tuberculosis transmission.

Here, we explored the possible links between the fitness of different drug-resistant *M tuberculosis* genotypes as reported from previous in-vitro experiments, with the corresponding transmission fitness in a defined epidemiological setting.

## Methods

### Study design and cohort selection

We performed whole-genome sequencing (WGS) of the available isolates from a retrospective cohort study of individuals routinely diagnosed with multidrug resistant (MDR) or rifampicin-resistant tuberculosis in primary care and hospitals Khayelitsha, Cape Town, South Africa, over a period of 10 years.^19^ Reporting of this study follows the STROME-ID guidance. Associated clinical data (eg, sex, previous tuberculosis treatment, and HIV infection) were retrieved from a prospectively maintained database from the Western Cape Provincial Health Data Centre.^20^

Khayelitsha is a poor, peri-urban subdistrict in Cape Town, South Africa. Among the population of approximately 400 000 people, antenatal HIV prevalence is 34% and tuberculosis case notification rate is 1600 per 100 000 people per year.^21^ HIV prevalence among people with tuberculosis is more than 70%.^21^ The estimated incidence of MDR or rifampicin-resistant-tuberculosis is 80 cases per 100 000 per year.^21^ Before 2011, only people with tuberculosis at a high risk for MDR or rifampicin-resistant tuberculosis were tested. Subsequently, all people with presumed tuberculosis were tested with Xpert MTB/RIF (Cepheid, Sunnyvale, CA, USA), with further drug susceptibility testing as per national guidance.^20^ All individuals diagnosed with rifampicin-resistant-tuberculosis were included in the observational cohort study, and those with linked, biobanked specimens included in this study.

The storage of MDR or rifampicin-resistant isolates in the biobank was approved by the Stellenbosch University ethics committee (N09/11/296), and linkage of WGS data to patient clinical data was approved by the University of Cape Town ethics committee (HREC 416/2014). Permission to access Western Cape Provincial Health Data Centre data was granted by the Western Cape Department of Health. There was no written informed consent from patients, because biobanked specimens were linked with routinely collected data with the data subsequently de-identified for analysis.

### WGS and sequence analysis

To identify the *M tuberculosis* complex lineage, genotypic resistance profiles, and infer the patient-to-patient transmission of *M tuberculosis* complex strains, we performed WGS of the available isolates from the study cohort. To detect drug resistance, we compared the mutations detected in each strain with an in-house catalogue of known drug-resistance-conferring mutations.^12^ Additionally, we analysed mutations that were not described in this catalogue, but were within genes associated with drug resistance.^6^ Mutations leading to a frameshift or premature stop codon in these genes were also considered as conferring drug resistance. Isolates in which no rifampicin-resistance-conferring mutations were detected were excluded from downstream analyses. Isolates that were predicted to be resistant to rifampicin but not to isoniazid were classified as rifampicin mono-resistant (RMR). Isolates resistant to at least rifampicin and isoniazid were classified as multidrug resistant. Multidrug resistant strains with additional resistance to fluoroquinolones and one of the injectable drugs were classified as extensively drug resistant, using the definition contemporary to the study period. To identify compensatory mutations, we collated a catalogue of putative compensatory mutations^12^ (appendix 1). We only considered high-confidence compensatory mutations, defined as those that independently evolved at least three times in a large-scale phylogenetic analysis.^12^ To identify the lineage of each strain, we compared the single nucleotide polymorphisms in each genome to a catalogue of lineage-defining single nucleotide polymorphisms.^22^ Details regarding the WGS of the isolates and the bioinformatic analysis, as well as quality control criteria, are detailed in appendix 2 (pp 1–3). Raw WGS data were deposited in the European Nucleotide Archive (accession numbers PRJEB45389 and PRJNA670836; appendix 3).

### Bayesian reconstruction of transmission trees

Details regarding the phylogenetic and transmission analysis are described in appendix 2 (pp 2–3). Briefly, to account for potential differences in substitution rates among the different *M tuberculosis* complex lineages,^23^ we built separated lineage-specific timed phylogenetic trees, specifying for each lineage the corresponding substitution rate as calculated previously.^23^ Then, transmission trees were inferred with TransPhylo (version 1.4.5) on the basis of the timed trees and the following model priors: the sampling proportion of our dataset (pi=0·4, based on the proportion of sequenced samples among diagnosed individuals and notification rates in this setting), within-host effective population size (population size × generation time=1·21, based on previous publications^24^), and basic reproductive number (off-*r*=1 and off-p=0·5). To test the robustness of our results to changes in the model priors, we performed sensitivity analyses by using a range of wide generation and sampling time γ distributions (appendix 2 p 12).

### Statistical analysis

All statistical analyses and figures were performed in R (version 4.1.3). Contingency tests were performed using Fisher's exact test. The difference in the number of drug-resistant-conferring mutations between groups was tested using two-sided Wilcoxon sum rank tests, given the non-normality of the data as tested with the Shapiro-Wilk test. In multivariable regression models, factors were included on the basis of univariate significance and presumed relevance on the basis of the literature. Individuals with missing data for the response variable were excluded when applicable. All models were corrected by individual age (as a continuous variable), sex, and HIV status. The significance threshold used was α=0·05 in all cases. Factors associated with the number of drug-resistance-conferring mutations were analysed in an adjusted multivariable Poisson regression. The phylogenetic multivariable logistic regression analyses of factors associated with MDR or rifampicin-resistant tuberculosis phenotypes and the transmission of these resistant diseases were performed using the package phylolm^25^ to correct for the underlying phylogenetic structure of the data. In this regression, cases diagnosed before 2016 were excluded to correct for the underestimation of transmitters at the end of the study period (see appendix 2 p 3).

### Role of the funding source

The funders of the study had no role in study design, data collection, data analysis, data interpretation, or writing of the report.

## Results

Between Jan 1, 2008, and Dec 31, 2017, 2161 individuals were diagnosed with MDR or rifampicin-resistant tuberculosis in Khayelitsha, Cape Town, South Africa. Whole-genome sequences that passed quality control were available for 1168 (54%) unique individual *M tuberculosis* isolates (appendix 2 p 13). Appendix 2 (p 14) shows the distribution of individual variables and the proportion of sequenced isolates available among each group. We found that individuals with missing data regarding disease category or culture-negative conversion, and individuals with extrapulmonary tuberculosis were less likely to have a sequenced isolate, although the differences between groups were small (appendix 2 pp 4, 14).

**Figure 1:**
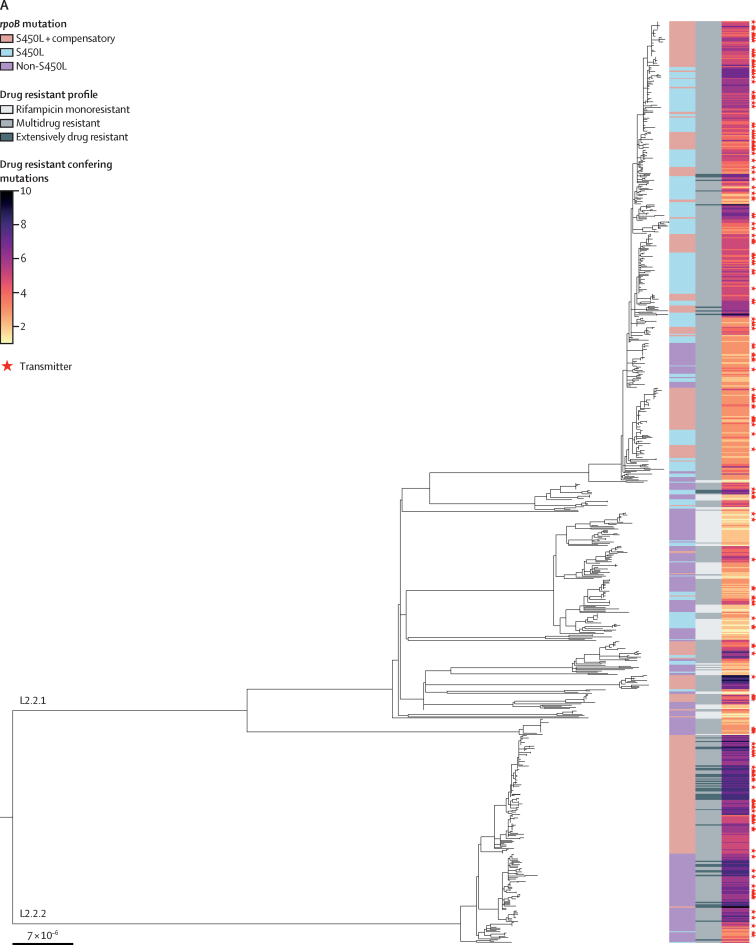

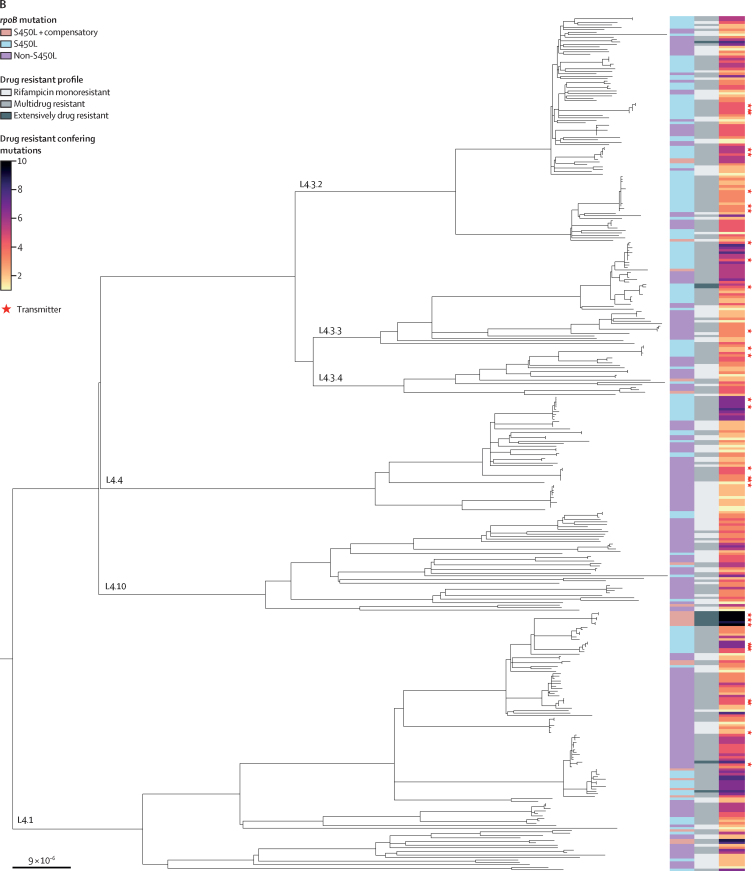
Maximum likelihood phylogenies for *Mycobacterium tuberculosis* complex lineage 2 and 4 strains The left side bars in blue, pink, and purple indicate the category of *rpoB* mutations (S450L or other) and whether the strain carried compensatory mutations. The next bars indicate, with different tones of grey, the drug-resistant profile category (rifampicin mono-resistant, multidrug resistant, or extensively drug resistant). The right side heatmap, in colours ranging from yellow to black, indicates the number of drug-resistance-conferring mutations. Strains marked with a red star were isolated from participants inferred to transmit multidrug-resistant or rifampicin-resistant tuberculosis with TransPhylo.

Of the 1168 *M tuberculosis* isolates with WGS data, the majority belonged to lineage 2 (785 [67·2%]), followed by lineage 4 (349 [29·9%]), lineage 3 (28 [2·4%]), and lineage 1 (six [0·5%]). Given the small numbers of lineages 1 and 3 isolates, we focused our other analyses on lineages 2 and 4. Based on our WGS data, 866 (74·1%) of strains were multidrug resistant, 243 (20·8%) were RMR, and 59 (5·1%) were extensively drug resistant. *rpoB* S450L was the most common rifampicin-resistance-conferring mutation (730 [62·5%]), followed by D435V (83 [7·1%]), H445Y (64 [5·5%]), L452P (49 [4·2%]), L430P (45 [3·9%]), H445D (44 [3·8%]), and D435Y (37 [3·2%]), as well as other less frequent mutations (appendix 2 p 5). 327 strains (28·0%) carried high-confidence compensatory mutations. Among compensated strains, 300 (91·7%) belonged to lineage 2, whereas 27 (8·3%) belonged to lineage 4, suggesting a significant association of lineage 2 with compensation (Fisher's exact test p<0·0001). We also observed a significant association of compensation with the mutation *rpoB* S450L (Fisher's exact test p<0·0001), with most compensated strains carrying this mutation (318 [97·2%]). Figure 1 shows the maximum-likelihood phylogenies for lineage 2 and lineage 4 strains, together with information on *rpoB* mutations, the drug-resistant profile, number of drug-resistant-conferring mutations, and whether the individual was inferred to have transmitted rifampicin-resistant tuberculosis.

We first looked for individual and bacterial factors associated with multidrug resistance as opposed to RMR. In a phylogenetic multivariable logistic regression (appendix 2 p 6), we found that age (adjusted odds ratio 1·03, 95% CI 1·01–1·05), earlier diagnosis date during the study period (1·07, 1·00–1·15), carrying the *rpoB* S450L mutation (4·80, 2·12–10·84), and additionally carrying a compensatory mutation (43·29, 6·15–304·73) were positively associated with multidrug resistance. Extrapulmonary tuberculosis (0·39, 0·19–0·79) and previous tuberculosis treatment episodes (0·79, 0·64–0·97) were negatively associated with multidrug resistance. Of note, the high odds ratios and wide CIs observed for compensated *rpoB* S450L were because of the fact that virtually all compensated strains were multidrug resistant (323 [98·8%]), and only four strains harbouring compensatory mutations were RMR.

**Figure 2:**
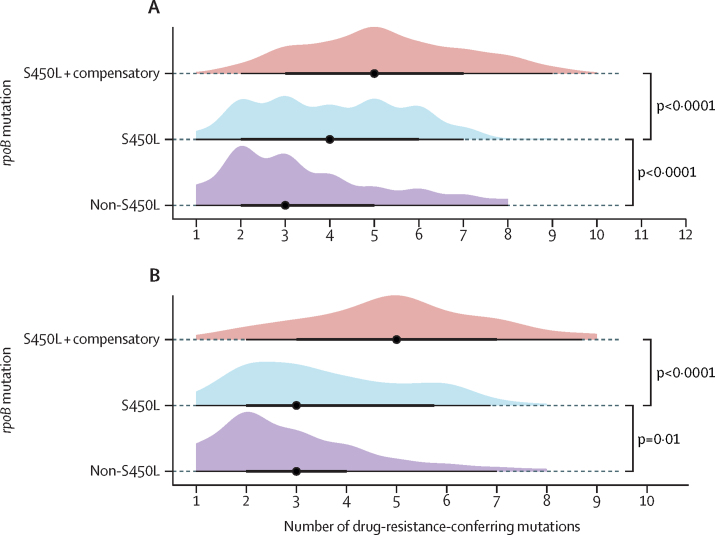
Number of drug-resistance-conferring mutations for different categories of *rpoB* mutations (non-S450L, S450L, and S450L with compensatory mutations) (A) Analysis for all lineage 2 and lineage 4 whole-genome sequences included in the study (n=1134). (B) Analysis for all unclustered isolates, plus the first sampled isolate of each cluster (n=355). p values correspond to the Wilcoxon rank sum test. The black dots represent the median, and the black bars represent the IQR.

The aforementioned results suggest that both the *rpoB* S450L mutation and additional compensatory mutations contribute to the amplification of drug resistance in *M tuberculosis*. In support of this notion, we observed that compensated *M tuberculosis* genotypes carried more drug-resistant-conferring mutations on average (figure 2A). However, association analyses might be confounded for highly transmitted strains, in which the same genotypes are counted repeatedly. To account for this potential bias, we grouped into clusters strains that are genetically closely related (appendix 2 p 2). Thus, we repeated our analysis including only the first sampled isolate of each cluster and observed the same effect (figure 2B). Moreover, using an adjusted multivariable Poisson regression correcting for genetic clustering and diagnosis date (appendix 2 p 7), we found that the number of drug-resistance-conferring mutations was higher for strains in transmission clusters (incidence rate ratio 1·30, 95% CI 1·20–1·42), strains carrying the *rpoB* S450L mutation (1·14, 1·06–1·22), and additionally carrying a compensatory mutation (1·38, 1·28–1·48). When repeating the multivariable regression analysis including only the first sampled isolate of each cluster, we found the same results (appendix 2 p 8).

**Figure 3:**
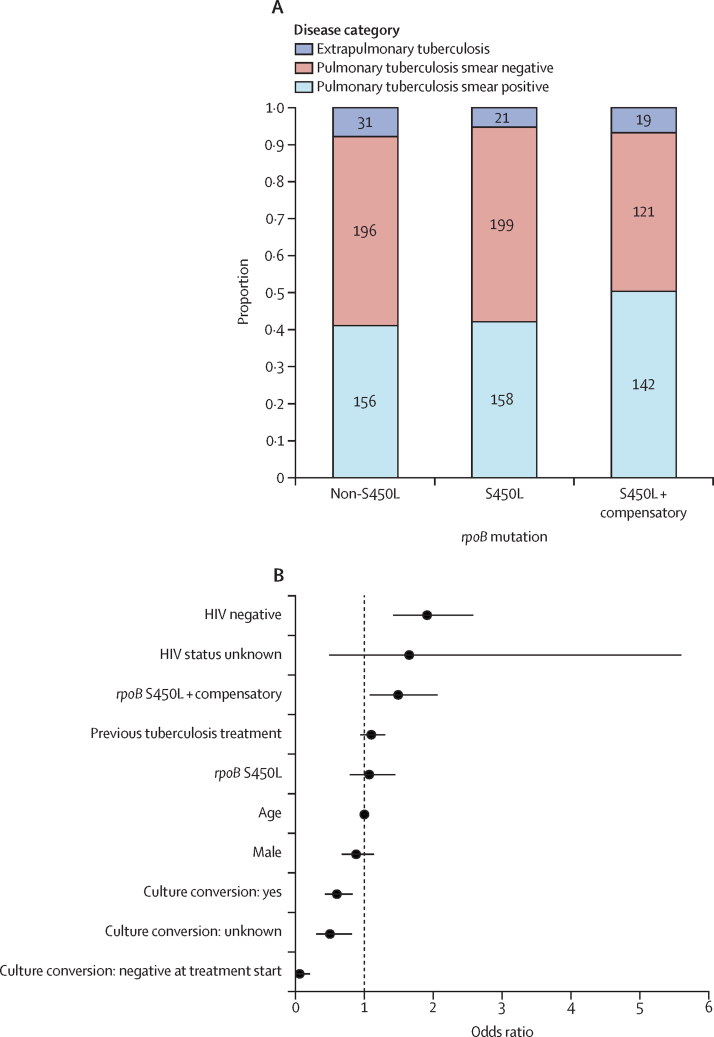
Factors associated with smear positivity (A) Bar plots with counts and proportion of different disease categories among groups with different *rpoB* mutations. Only data for participants with known disease category are shown. (B) Multivariable logistic regression of factors associated with smear-positive microscopy.

We hypothesised that the higher number of drug-resistant-conferring mutations among high-fitness genotypes might be the consequence of a higher replication within hosts. To explore this, we performed a multivariable logistic regression analysis using smear positivity as the outcome and as a proxy for in vivo replication. Being HIV negative (adjusted odds ratio 1·91, 95% CI 1·42–2·58), and being infected with an *rpoB* S450L compensated strain (1·49, 1·08–2·06) were positively associated with smear positivity, whereas culture-negative conversion was negatively associated (figure 3; appendix 2 p 9).

**Figure 4:**
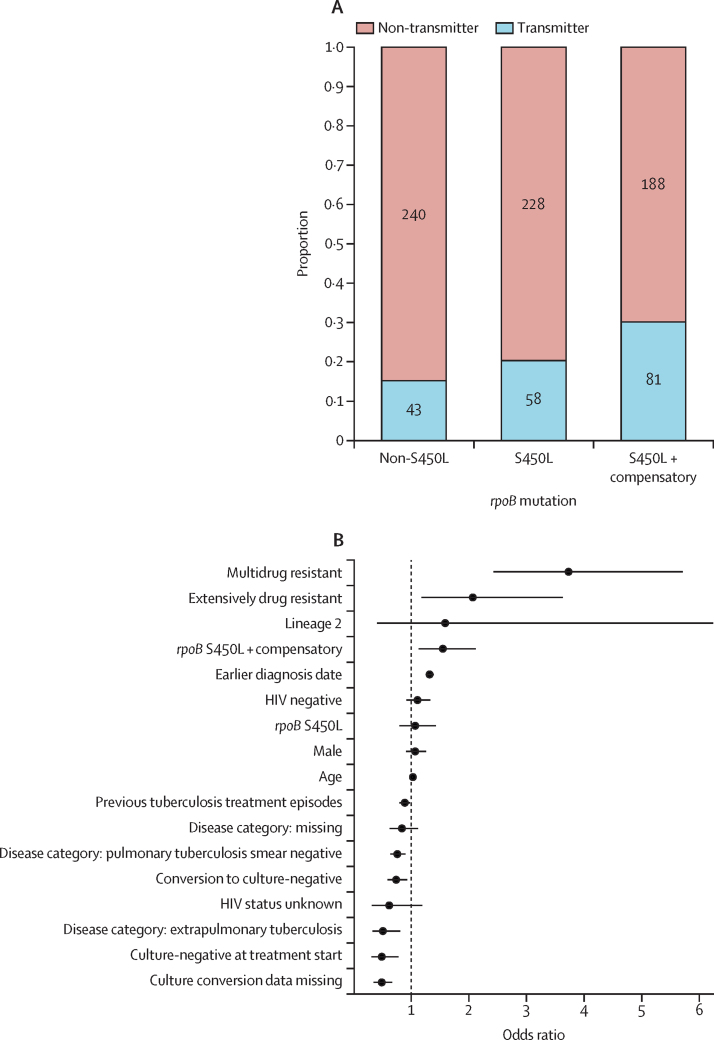
Factors associated with transmission of multidrug-resistant and rifampicin-resistant tuberculosis (A) Bar plots with counts and proportion of transmitters among groups with different *rpoB* mutations. (B) Phylogenetic multivariable logistic regression of factors associated with being a transmitter of multidrug-resistant and rifampicin-resistant tuberculosis.

To find factors driving the transmission of MDR or rifampicin-resistant tuberculosis in Khayelitsha, we used TransPhylo to identify individuals who probably transmitted the disease in our study population. Among the 838 individuals diagnosed before 2016 and included in the transmission analysis (appendix 2 p 3), 182 (21·7%; figure 4) were identified as transmitters of MDR or rifampicin-resistant tuberculosis. Overall, the proportion of transmitters was higher among individuals carrying a strain with the *rpoB* S450L mutation, and even higher if the strain carried an additional compensatory mutation (figure 4A). To find factors associated with being a transmitter, we performed a multivariable logistic regression adjusted by individual factors and the phylogenetic structure using phylolm (figure 4B; appendix 2 pp 10–11). We found that previous tuberculosis treatment (adjusted odds ratio 0·89, 95% CI 0·80–0·98), smear-negative microscopy (0·76, 0·64–0·90), extrapulmonary disease (0·51, 0·33–0·81), and presenting a negative culture before treatment (0·49, 0·35–0·67) or converting to culture negative after treatment (0·74, 0·59–0·93) were negatively associated with transmitting MDR or rifampicin-resistant tuberculosis, whereas carrying an MDR (3·73, 2·43–5·71) or extensively drug-resistant (2·07, 1·18–3·63) strain compared with an RMR strain, carrying *rpoB* S450L and a compensatory mutation (1·55, 1·13–2·12), an earlier diagnosis year during the study (1·32, 1·25–1·39), and individual age (1·03, 1·02–1·03) were positively associated (figure 4B; appendix 2 pp 10–11). Our sensitivity analyses indicated that the results obtained with TransPhylo (appendix 2 pp 15–16) and in the multivariable regression analysis (appendix 4) were robust to changes in the model priors.

## Discussion

We studied the in vivo fitness of MDR or rifampicin-resistant *M tuberculosis* genotypes in a population in Khayelitsha, Cape Town, South Africa, a high MDR and rifampicin-resistant tuberculosis incidence setting, over a period of 10 years. Our results showed that high-fitness genotypes were associated with a higher number of drug-resistant-conferring mutations and positive smear microscopy, which together suggest a higher replicative capacity within the human host. Moreover, we found that higher levels of drug-resistance-conferring, positive-smear microscopy and compensatory evolution independently contributed to the transmission fitness (between-host fitness) of MDR or rifampicin-resistant *M tuberculosis* in this setting.

The potential of *M tuberculosis* to evolve drug-resistant-conferring mutations under antibiotic treatment pressure directly depends on the replicative capacity within a host. This notion implies that, hypothetically, the lower the fitness cost associated with a particular drug-resistant-conferring mutation, the higher the probability of acquiring novel mutations conferring drug resistance to additional drugs. Our results support this idea. We observed that rifampicin-resistant strains carrying the low fitness cost mutation *rpoB* S450L were associated with a higher number of drug-resistant-conferring mutations, and that the number of drug-resistant-conferring mutations further increased when the strain also carried compensatory mutations. Compensated strains have been previously found to carry more drug-resistant-conferring mutations in other settings.^10^, ^13^ However, it has been argued that this previous observation might have been because of the confounding effect of genetic clustering and the length of time that a strain has been circulating in the population.^14^ There are several reasons to think that our observations are unlikely to stem from such confounding effects. First, we corrected our multivariable regression analysis for genetic clustering, and we also showed that the same results were obtained when conducting a stringent analysis including only the first sampled isolate of each cluster. Second, the number of drug-resistant-conferring mutations is expected to depend on the length of time that a particular genotype has been circulating (and thus evolving). However, this would not explain why some *rpoB* mutations (S450L or non-S450L) are associated with a higher number of drug-resistant-conferring mutations. Additionally, we found high-fitness drug-resistant genotypes to be associated with sputum smear-positivity, which further supports the notion that genotypes with high-fitness mutations (as measured in vitro), might also replicate better within the host. Another question is whether lineage 2 strains are also more prone to acquire drug-resistant-conferring mutations. One study showed that the risk of drug resistance acquisition is higher for lineage 2 compared with lineage 4 strains.^26^ In our study, we observed that, for strains that were already resistant to rifampicin, the bacterial lineage did not seem to play any role in the further amplification of drug resistance (appendix 2 pp 7–8). Our results indicate that for rifampicin-resistant strains, the relative fitness cost of different rifampicin-resistance and compensatory mutations have a stronger effect on drug resistance amplification than the strain lineage.

We also found that bacterial factors play a direct role in the transmission of MDR or rifampicin-resistant tuberculosis in Khayelitsha. Remarkably, these factors were interconnected, and seem to affect the transmission of rifampicin-resistant tuberculosis in a synergistic manner. Genotypes carrying both the low fitness cost mutation *rpoB* S450L and compensatory mutations showed an increased likelihood of MDR or rifampicin-resistant tuberculosis transmission. At the same time, these genotypes showed an increased number of drug-resistant-conferring mutations and were more likely to be multidrug resistant (as opposed to RMR) and to present smear-positive microscopy. In turn, individuals carrying MDR strains and presenting smear-positive microscopy were also more likely to transmit the disease than rifampicin monoresistant strains and smear-negative microscopy. Overall, other factors associated with the transmission of rifampicin-resistant tuberculosis in this study were in line with the literature. Several studies have shown the MDR tuberculosis epidemic to be driven mainly by transmission.^1^, ^12^, ^27^, ^28^ We show that in Khayelitsha, MDR and extensively drug-resistant genotypes have a higher risk of transmission compared with RMR genotypes. This finding could be explained by shorter infectious periods for RMR genotypes, given that the standard first-line treatment regimens containing isoniazid are efficacious in individuals carrying RMR strains. The association of smear-positivity with transmission was not surprising, because sputum-smear microscopy has been commonly identified as a predictor of transmissibility.^29^ Similarly, other factors that are thought to be linked to a lower infectiousness, such as extrapulmonary tuberculosis and culture-negative conversion, were also associated with a lower risk of MDR or rifampicin-resistant-tuberculosis transmission in our analysis, further supporting the validity of our findings. Most importantly, we found that the fitness cost of rifampicin resistance, as previously measured in vitro, and particularly compensatory evolution have a direct effect on the transmission of MDR or rifampicin-resistant tuberculosis, independently of other bacterial and patient factors.

In contrast with classic molecular epidemiological studies based on genetic clustering, we used TransPhylo to answer the question of who transmitted to whom. Although previous evaluations have shown that TransPhylo has a good performance identifying tuberculosis transmitters, this method still presents some limitations.^30^ One of these is that transmitters might not be identified as such if the individuals they transmitted to are unsampled. In our study, we applied two correcting strategies to account for such a potential bias: we limited the multivariable analyses to individuals diagnosed before the last 2 years of the study (before 2016, thus allowing an incubation time of 2 years), and adjusted the regression models by diagnosis date. Another potential pitfall when performing Bayesian transmission tree reconstruction is the choice of the parameter priors that define the model of transmission, as well as to what extent the results of the analysis depend on such priors. To account for this, we carried out a series of sensitivity analyses using a range of different model priors (appendix 2 p 12). Our sensitivity analyses showed that: (1) the posterior distributions of the generation and sampling times calculated by TransPhylo were different from the prior distributions (appendix 2 p 15); (2) the number of transmitters was not dependent on the model priors (appendix 2 p 16); and (3) the main results of the study were robust to changes in the model priors (appendix 4).

Whether our results can be generalised to different settings warrants future research. However, taken together, the available evidence suggests that the effect of compensatory evolution in the emergence and spread of drug-resistant tuberculosis is not limited to Khayelitsha. Our results are relevant for the control of drug-resistant tuberculosis worldwide, given the new treatment regimens based on novel drugs that are increasingly being rolled out to treat MDR or rifampicin-resistant tuberculosis. We need to design appropriate surveillance and intervention strategies to deliver the best possible treatment for individuals and prevent the emergence and spread of highly drug-resistant and transmissible *M tuberculosis* strains.

## Data sharing

All sequencing data are available online in the European Nucleotide Archive repository under accession numbers PRJEB45389 and PRJNA670836. Accession numbers for the samples analysed in this study and associated metadata can be found in appendix 3.

## Declaration of interest

RMW acknowledges baseline support from the South African Medical Research Council. All other authors declare no competing interests.
