# Supplementary material for: Effect of compensatory evolution in the emergence and transmission of rifampicin-resistant Mycobacterium tuberculosis in Cape Town, South Africa: a genomic epidemiology study
Source: Lancet Microbe. 2023 Jul;4(7):e506–15. doi: 10.1016/S2666-5247(23)00110-6 (PMC10319636; doi:10.1016/S2666-5247(23)00110-6)
Supplement: Supplementary appendix 2 [file mmc2.pdf]

THE LANCET
Microbe

Supplementary appendix 2

This appendix formed part of the original submission and has been peer reviewed. We post it as supplied by the authors.

Supplement to: Goig GA, Menardo F, Salaam-Dreyer Z, et al. Effect of compensatory evolution in the emergence and transmission of rifampicin-resistant *Mycobacterium tuberculosis* in Cape Town, South Africa: a genomic epidemiology study. *Lancet Microbe* 2023; published online June 6. https://doi.org/10.1016/S2666-5247(23)00110-6.

Supplementary Methods

Whole genome sequencing methods

Available isolates were recultured for DNA extraction and sequencing. WGS was done on libraries prepared from purified genomic DNA using Illumina Nextera XT library (Illumina, San Diego, CA, USA) and NEBNext Ultra TM II FS DNA Library Prep Kits (New England Biolabs, Ipswich, MA, USA). Sequencing was done using the Illumina HiSeq 2500 or NextSeq 500 platforms. The minimum quality criteria for sequencing data were minimum sequencing depth of 20×, 90% genomic coverage, contamination with non-tuberculous mycobacteria DNA lower than 1%[1] and a ratio of variable to fixed SNPs lower than 0·2. Trimmomatic (v0.33)[2] was used to clip adapters and filter for quality, whereby <20-bp reads were discarded. Overlapping paired-end reads were merged with SeqPrep[3]. The resulting reads were mapped to a reconstructed ancestral genome of the MTBC (available at Zenodo 10·5281/zenodo.3497110) with BWA (v0.7.12)[4]; duplicate reads were marked and removed with Picard (v2.1.1)[5]. To enhance mapping of reads near indels, local realignments were performed with GATK (v3.4.0)[6]. Pileups were generated with SAMtools (v1.2)[7], and single-nucleotide variants (SNPs) were subsequently called with VarScan (v2.4.1)[8], applying the following thresholds: minimum base quality of 20 and minimum read depth 7× at a given position. SNPs were called if at least five reads supported the alternative allele without strand bias. A given SNP was considered fixed if its frequency reached 90%, and a position was called as ancestral if the frequency was below 10%. The effect of the SNPs was inferred using SnpEff (v4.11)[9] using the *M. tuberculosis* H37Rv reference annotation (NC_000962.3). Additionally, we only included in the analysis samples that fulfilled the following criteria. Whenever more than one isolate was available for the same TB patient, the first isolate was used. Isolates with evidence of polyclonal infections were excluded from the analysis. Patients showing different MTBC strains in longitudinal samples were also excluded, to avoid potential confounding effects when inferring transmission.

Bayesian reconstruction of transmission trees

To account for potential differences in substitution rates among the different MTBC*s* lineages[10], we built separated lineage-specific timed phylogenetic trees specifying for eac lineage the corresponding substitution rate as calculated previously[10]. For each lineage, we obtained a pseudo-alignment of polymorphic positions by concatenating all high-quality SNPs in the dataset, excluding resistance-associated positions. Non-fixed SNPs (<90% allele frequency) or positions covered with less than 7 reads were encoded as "X" in the alignment. Positions in the alignment where more than 10% of sequences had an X, were discarded. SNP alignments were used to infer maximum-likelihood phylogenies for each lineage using IQ-TREE 2[11] with the general time-reversible model of sequence evolution, indicating the invariant sites of each nucleotide. Phylogenetic trees were dated using LSD2[12] specifying for each lineage the corresponding substitution rate as calculated previously[13]. The substitution rates used were 1E-7 for lineage 2, and 5.9E-8 for lineage 4. TransPhylo analyses were performed individually for each cluster of patients with a close phylogenetic link. Transmission clusters are usually defined based on a maximum SNP distance threshold between bacterial genomes. However, this approach can introduce a bias due to the different substitution rates across the MTBC lineages[14]. To circumvent this issue, we defined clusters based on the time to the most recent common ancestor. Strains that shared a common ancestor at most 15 years ago, were considered within the same transmission cluster. From the original timed tree of each lineage, the subtree of each cluster was obtained and analyzed with TransPhylo. We analyzed all subtrees of at least four patients in the same TransPhylo run, using the *infer_multittree_share_param* function. TransPhylo runs were carried out separately for the different lineages in the dataset.

TransPhylo requires the user to specify model priors that describe the process of pathogen transmission, evolution and sampling. Generation time (the time that happens since one host becomes infected until he/she becomes infectious) and the sampling time (the time that elapses since one host becomes infected until he/she is diagnosed) are of particular relevance when inferring transmission of *M. tuberculosis*, given the long and variable latency periods of this pathogen. To account for such variability, we specified generation and sampling times with wide gamma distributions (shape 1.5 and scale 1;

Supplementary figure 1). Additionally, we specified parameters for the sampling proportion of our dataset (pi=0.4; based on the proportion of sequenced samples among diagnosed cases and notification rates in this setting), within-host effective population size (NeG=1.21; based on previous publications[15]) and basic reproductive number (off-r=1, off-p=0.5), allowing the algorithm to update the value through the algorithm run. Each TransPhylo analysis was run for one million iterations with a thinning of 100 and a burn-in of the initial 50% transmission trees, checking convergence of the trace plots and ensuring effective sample sizes for all parameters of at least 200. Therefore, for each transmission cluster, 5,000 posterior transmission trees were inferred by TransPhylo and further analyzed. Patients were classified as transmitters of TB whenever they were inferred to transmit to at least one other patient in at least half of the posterior transmission trees. Patients who transmitted at the end of the study period could not be identified as such, given that any individuals they may have infected would only have potentially developed TB disease after the end of the study, thus remaining unsampled. To account for this limitation, we used TransPhylo to analyze the complete dataset, but excluded from the downstream transmission analysis patients diagnosed after December 2016. Given the relevance of the generation and sampling time parameters, we tested the robustness of the TransPhylo inference to changes in these parameters. We performed sensitivity analyses by using a range of wide generation and sampling time gamma distributions with a mean of the latency period of 1, 1.5, 2, 2.5, 3 and 3.5 years (Supplementary Figure 1).

Supplementary Results

Supplementary Table 1: Results of a multivariable logistic regression of patient factors associated with having an isolate with WGS data. PTB stands for pulmonary tuberculosis, EPTB stands for extrapulmonary tuberculosis and Tx stands for treatment.

	Sample size (n = 2,041)	Multivariable aOR (95% CI; n=1134)	p-value
**Age**		0.99 (0.98-1.00)	0.22
**Sex**			
Female	991	1 (ref)	
Male	1050	0.97 (0-78 – 1.21)	0.81
**HIV status**			
Positive	1505	1 (ref)	
Negative	502	1.17 (0.90 – 1.53)	0.25
Unknown	34	1.25 (0.45 – 3.48)	0.66
**Disease category**			
PTB smear-positive	910	1 (ref)	
PTB smear-negative	836	0.96 (0.76 – 1.22)	0.74
EPTB	163	**0.53 (0.35 – 0.80)**	**0.01**
Missing data	132	**0.39 (0.24 – 0.63)**	**0.01**
**Previous TB treatment**			
Previous TB treatment	1354	1 (ref)	
No previous TB treatment	650	0.90 (0.71 – 1.14)	0.40
Unknown	37	0.68 (0.26 – 1.66)	0.40
**Sputum culture negative conversion**			
No conversion	361	1 (ref)	
Culture-negative converted	1295	1.08 (0.80 – 1.45)	0.63
Culture negative at Tx start	81	0.57 (0.30 – 1.07)	0.09
Unknown	304	**0.58 (0.39 – 0.86)**	**0.01**
**Diagnosis date**		1.00 (1.00 – 1.00)	0.95

Supplementary Table 2: Number of isolates in which the different RpoB mutations were observed.

RpoB mutation	Count	Percentage (n=1,168)	RpoB mutation	Count	Percentage (n=1,168)
S450L	730	62.50 %	Q432L	3	0.26 %
D435V	86	7.36 %	V170F	2	0.17 %
H445Y	66	5.65 %	F433_M434del	2	0.17 %
L452P	53	4.54 %	Q436del	2	0.17 %
L430P	50	4.28 %	Q436_N437del	2	0.17 %
H445D	48	4.11 %	I491L	1	0.09 %
D435Y	41	3.51 %	Q432_D435delinsH	1	0.09 %
H445L	16	1.37 %	D435_Q436del	1	0.09 %
D435F	13	1.11 %	MD434IY	1	0.09 %
H445N	13	1.11 %	H445_K446del	1	0.09 %
S450W	10	0.86 %	S428T	1	0.09 %
S450F	8	0.68 %	Q432_F433del	1	0.09 %
H445R	6	0.51 %	S450Y	1	0.09 %
D435G	6	0.51 %	L430R	1	0.09 %
H445R	6	0.51 %	Q492L	1	0.09 %
S441L	5	0.43 %	R448K	1	0.09 %
S441L	5	0.43 %	H445G	1	0.09 %
Q432P	4	0.34 %	D435A	1	0.09 %
Q436del	4	0.34 %	LS430PT	1	0.09 %
Q432P	4	0.34 %	M434_D435del	1	0.09 %
Q432L	3	0.26 %	T427A	1	0.09 %
Q432K	3	0.26 %	D435del	1	0.09 %
S431G	3	0.26 %	I491V	1	0.09 %
I491F	3	0.26 %	N438del	1	0.09 %
Q432K	3	0.26 %			

Supplementary Table 3: Phylogenetic multivariable logistic regression of factors associated with being MDR as opposed to RMR. PTB stands for pulmonary tuberculosis, EPTB stands for extrapulmonary tuberculosis.

	Sample size (n = 1,134)	Multivariable aOR (95% CI; n=1,134)	p-value
**Lineage**			
4	349	1 (ref)	
2	785	1.79 (0.70-4.59)	0.23
**RpoB mutation**			
non-S450L	426	1 (ref)	
S450L	390	**4.80 (2.12-10.84)**	**<0.0001**
S450L+Compensatory	318	**43.29 (6.15-304.73)**	**<0.0001**
**Previous TB treatment episodes**		**0.79 (0.64-0.97)**	**0.02**
**Age**		**1.03 (1.01-1.05)**	**0.01**
**Sex**			
Female	550	1 (ref)	
Male	584	1.44 (0.99-2.11)	0.06
**HIV**			
Positive	806	1 (ref)	
Negative	300	1.38 (0.90-2.12)	0.14
Missing data	28	1.91 (0.19-19.05)	0.58
**Diagnosis date** In years since end of the study period (2018)		1.07 (1.00-1.15)	**0.04**
**Disease category**			
PTB smear-positive	447	1 (ref)	
PTB smear-negative	497	1.14 (0.81-1.62)	0.45
EPTB	67	**0.39 (0.19-0.79)**	**0.01**
Missing data	123	0.93 (0.54-1.59)	0.79

Supplementary Table 4: Multivariable Poisson regression on the number of DR-conferring mutations corrected by genetic clustering and diagnosis date. PTB stands for pulmonary tuberculosis, EPTB stands for extrapulmonary tuberculosis.

	Sample size (n = 1,134)	Multivariable IRR (95% CI; n=1,134)	p-value
**Lineage**			
4	349	1 (ref)	
2	785	1.05 (0.97-1.13)	0.23
**Genetic clustering**			
No	257	1 (ref)	
Yes	911	**1.30 (1.20-1.42)**	**<0.0001**
**RpoB mutation**			
non-S450L	426	1 (ref)	
S450L	390	**1.14 (1.06 – 1.22)**	**0.01**
S450L+Compensatory	318	**1.38 (1.28 – 1.48)**	**<0.0001**
**Previous TB treatment episodes**	1134	1.03 (0.99 – 1.06)	0.12
**Age**	1134	1.00 (0.99 – 1.00)	0.62
**Sex**			
Female	550	1 (ref)	
Male	584	0.96 (0.90 – 1.02)	0.15
**Disease category**			
PTB smear-positive	447	1 (ref)	
PTB smear-negative	497	0.97 (0.91-1.03)	0.36
EPTB	67	0.89 (0.78-1.02)	0.10
Missing data	123	1.05 (0.95-1.16)	0.32
**HIV**			
Positive	806	1 (ref)	
Negative	300	1.05 (0.98 – 1.13)	0.13
Missing data	28	0.97 (0.75 – 1.23)	0.78

Supplementary Table 5: Multivariable Poisson regression on the number of DR-conferring mutations corrected by genetic clustering and diagnosis date. For clustered strains, only the first isolate of the cluster was analysed. PTB stands for pulmonary tuberculosis, EPTB stands for extrapulmonary tuberculosis.

	Sample size (n = 355)	Multivariable IRR (95% CI)	p-value
**Lineage**			
4	177	1 (ref)	
2	178	0.94 (0.82-1.07)	0.35
**Genetic clustering**			
No	246	1 (ref)	
Yes	109	**1.40 (1.22-1.60)**	**<0.0001**
**RpoB mutation**			
non-S450L	190	1 (ref)	
S450L	117	**1.18 (1.03 – 1.34)**	**0.01**
S450L+Compensatory	48	**1.64 (1.40 – 1.91)**	**<0.0001**
**Previous TB treatment episodes**	355	0.99 (0.92 – 1.06)	0.78
**Age**	355	1.00 (1.00 – 1.01)	0.51
**Sex**			
Female	171	1 (ref)	
Male	184	1.06 (0.95 – 1.19)	0.29
**Disease category**			
PTB smear-positive	125	1 (ref)	
PTB smear-negative	154	0.89 (0.78-1.01)	0.07
EPTB	31	0.84 (0.66-1.05)	0.13
Missing data	45	0.97 (0.80-1.17)	0.73
**HIV**			
Positive	269	1 (ref)	
Negative	78	1.05 (0.91 – 1.21)	0.48
Missing data	8	1.24 (0.67 – 2.07)	0.46
**Diagnosis date**	355	1.01 (0.99 – 1.03)	0.53

Supplementary Table 6: Multivariable logistic regression of factors associated to smear positivity. PTB stands for pulmonary tuberculosis, EPTB stands for extrapulmonary tuberculosis: Tx stands for treatment.

	Sample size (n = 1,134)	Multivariable aOR (95% CI; n=1,134)	p-value
**RpoB mutation**			
non-S450L	426	1 (ref)	
S450L	390	1.07 (0.79 -1.45)	0.93
S450L+Compensatory	318	**1.49 (1.08 – 2.06)**	**0.02**
**Previous TB treatment episodes** (continuous)		1.10 (0.94-1.30)	0.23
**Age** (continuous)		1.00 (0.99-1.01)	0.62
**Sex**			
Female	550	1 (ref)	
Male	584	0.88 (0.67- 1.14)	0.32
**Negative culture conversion**			
No culture conversion after Tx	205	1 (ref)	
Culture conversion after Tx	753	**0.60 (0.43-0.83)**	**0.01**
Culture negative at Tx start	36	**0.06 (0.01-0.21)**	**0.01**
Unknown	140	**0.50 (0.30-0.82)**	**0.01**
**HIV**			
Positive	806	1 (ref)	
Negative	300	**1.91 (1.42 – 2.58)**	**<0.0001**
Unknown	28	1.65 (0.49 – 5.60)	0.41

Supplementary Table 7: Phylogenetic multivariable logistic regression analysis of factors associated with being a transmitter of DR-TB. PTB stands for pulmonary tuberculosis, EPTB stands for extrapulmonary tuberculosis, Tx stands for treatment.

	Sample size (n = 838)	Multivariable aOR (95% CI)	p-value
**Lineage**			
4	232	1 (ref)	
2	606	1.59 (0.41-6.24)	0.50
**DR profile**			
RMR	137	1 (ref)	
MDR	652	**3.73 (2.43-5.71)**	**<0.0001**
XDR	49	**2.07 (1.18-3.63)**	**0.01**
**RpoB mutation**			
non-S450L	283	1 (ref)	
S450L	286	1.07 (0.80-1.43)	0.65
S450L+Compensatory	269	**1.55 (1.13-2.12)**	**0.01**
**Previous TB treatment episodes**		**0.89 (0.80-0.98)**	**0.02**
**Age**		**1.03 (1.02-1.03)**	**0.01**
**Sex**			
Female	409	1 (ref)	
Male	429	1.07 (0.91-1.26)	0.43
**HIV**			
Positive	596	1 (ref)	
Negative	220	1.11 (0.92-1.33)	0.27
Missing data	22	0.62 (0.32-1.19)	0.15
**Diagnosis date**			
In years since end of the study period		**1.32 (1.25-1.39)**	**<0.0001**

(Continues in the next page)

	Sample size (n = 838)	Multivariable aOR (95% CI)	p-value
**Disease category**			
PTB smear-positive	338	1 (ref)	
PTB smear-negative	363	**0.76 (0.64-0.90)**	**0.01**
EPTB	51	**0.51 (0.33-0.81)**	**0.01**
Missing data	86	0.84 (0.63-1.12)	0.23
**Negative culture conversion**			
No culture conversion after Tx	162	1 (ref)	
Culture conversion after Tx	536	**0.74 (0.59-0.93)**	**0.01**
Culture negative at Tx start	28	**0.49 (0.31-0.78)**	**0.01**
Unknown	112	**0.49 (0.35-0.67)**	**0.01**

Supplementary Figures

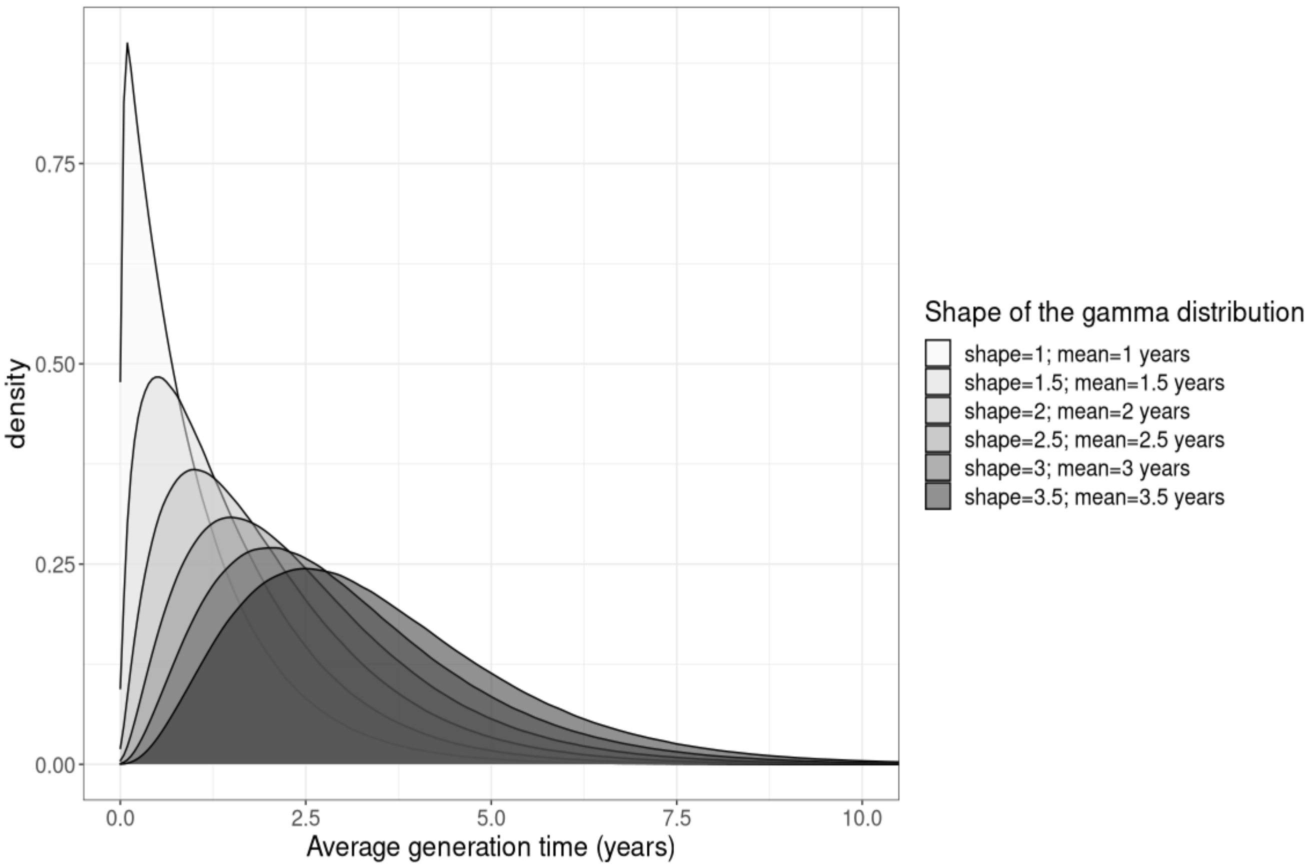

Supplementary Figure 1: Gamma distributions tested for the generation and sampling times in the sensitivity analysis. Results through the manuscript correspond to a gamma distribution with shape 1.5 and scale 1.

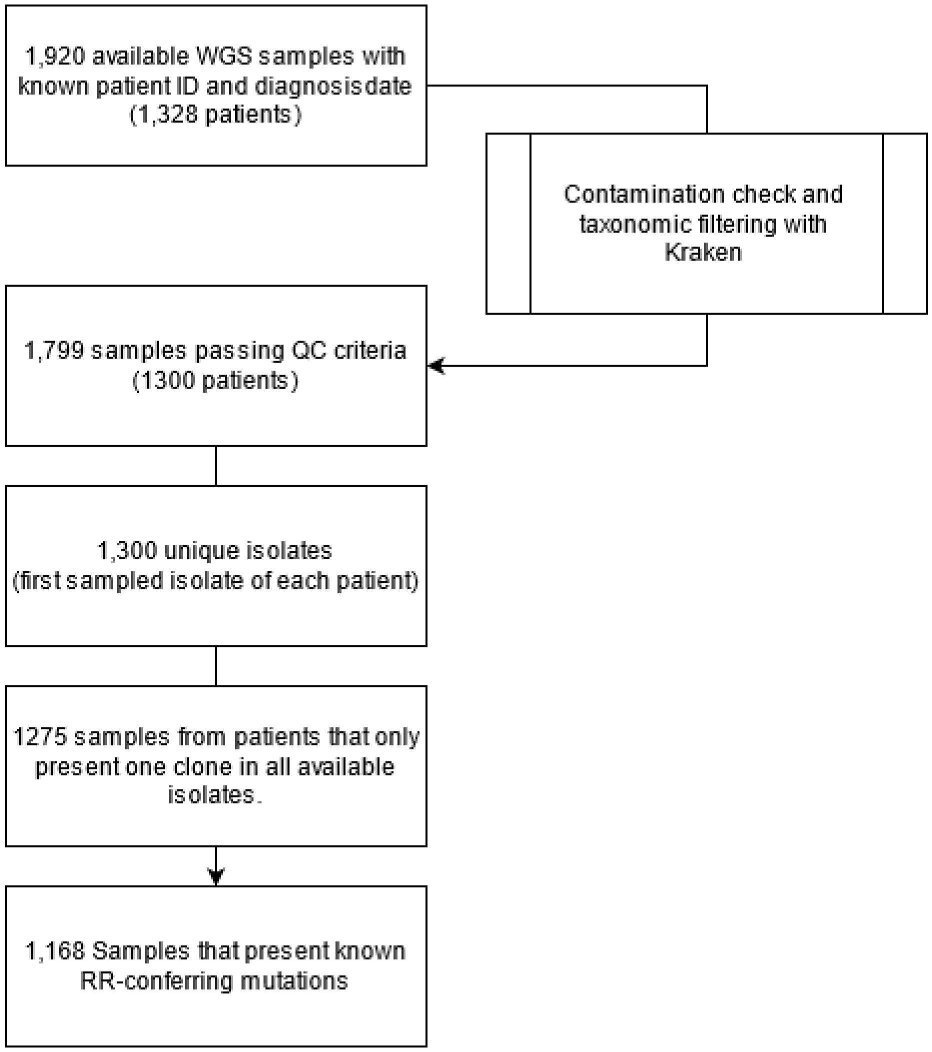

Supplementary Figure 2: Flow diagram of samples analyzed in this study. Quality control (QC) criteria were: genomic coverage at 7X depth greater than 90%, mean sequencing depth greater than 20X, mixed/fixed SNPs ratio lower than 0.2, and proportion of non-tuberculous mycobacteria reads lower than 1%. Additionally, we discarded samples from patients that showed different clones (>50 SNPs apart) in longitudinal samples (n=25). We only included in downstream analyses those genomes carrying rifampicin resistance (RR)-conferring mutations.

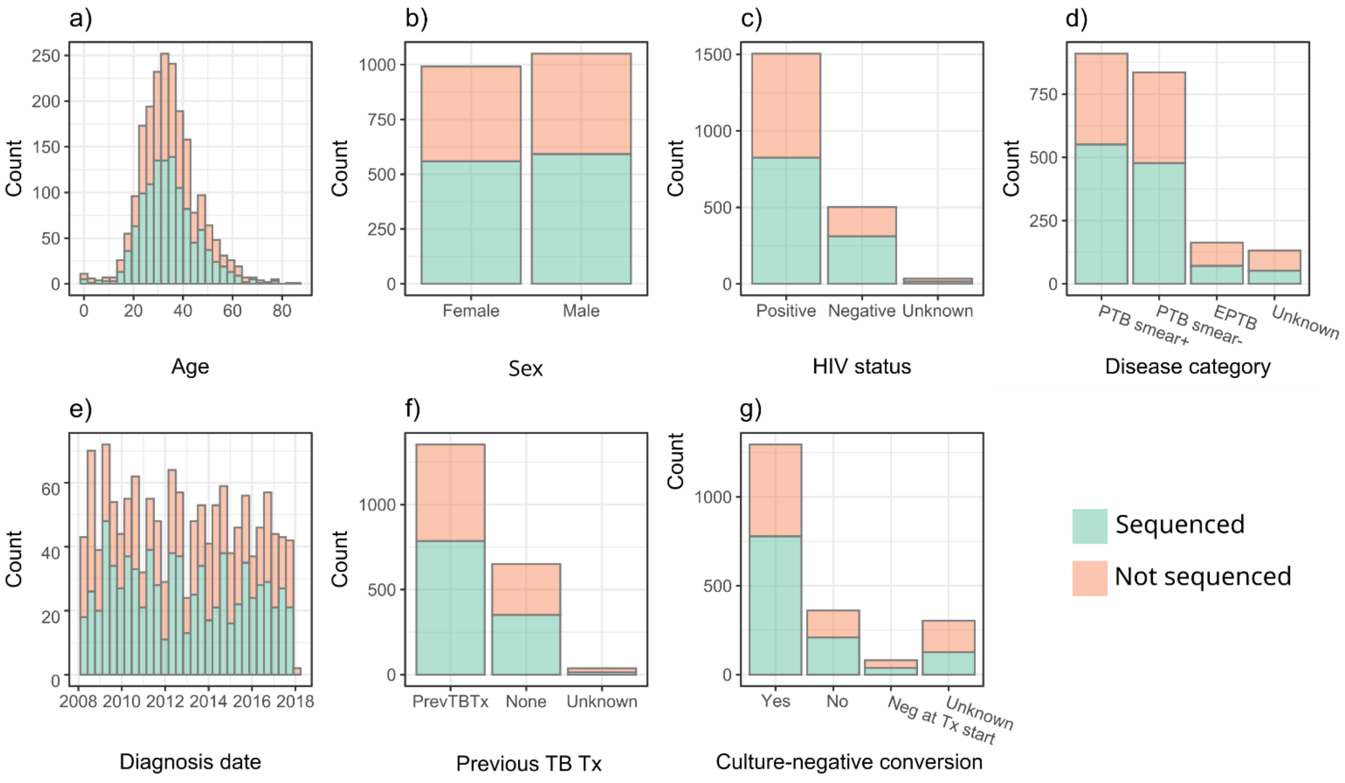

Supplementary Figure 3: Distribution of a) patient age, b) sex, c) HIV status, d) disease category (PTB is pulmonary tuberculosis; EPTB is extrapulmonary tuberculosis; Tx is treatment), e) diagnosis date, f) previous TB treatment episodes and g) sputum culture-negative conversion. Patients with sequenced isolates analyzed in this study are represented in green. Patients with missing data regarding disease category and culture conversion, and patients with EPTB were less likely to have an isolate sequenced (Supplementarty Table 1).

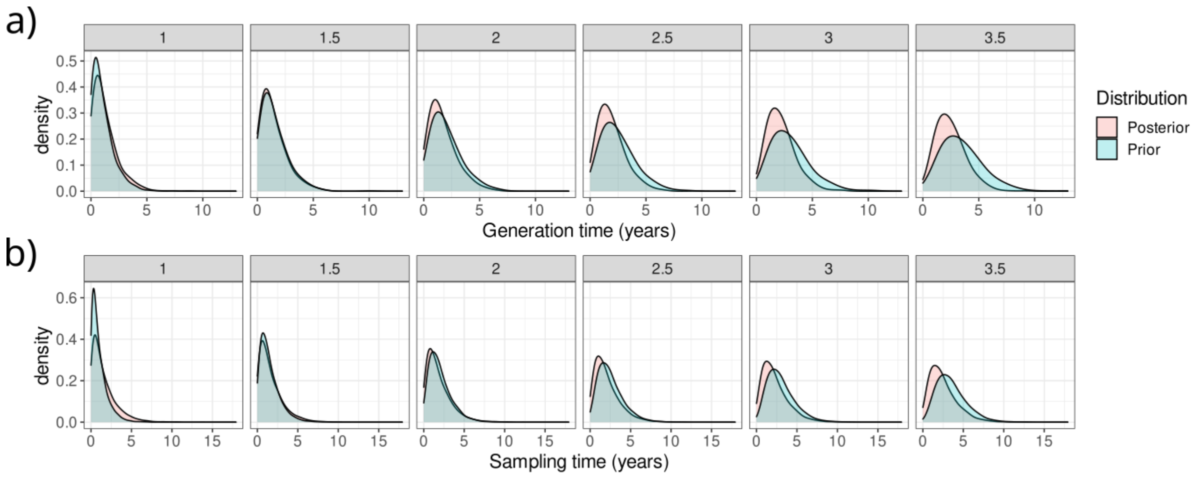

Supplementary Figure 4: Prior and posterior distributions for the different generation and sampling times used in the TransPhylo sensitivity analysis. Each panel correspond to a different gamma distribution prior (scale 1 and shapes 1, 1.5, 2, 2.5, 3 and 3.5 respectively). Panel a) corresponds to the distributions of the generation time; panel b) corresponds to the distributions of the sampling time.

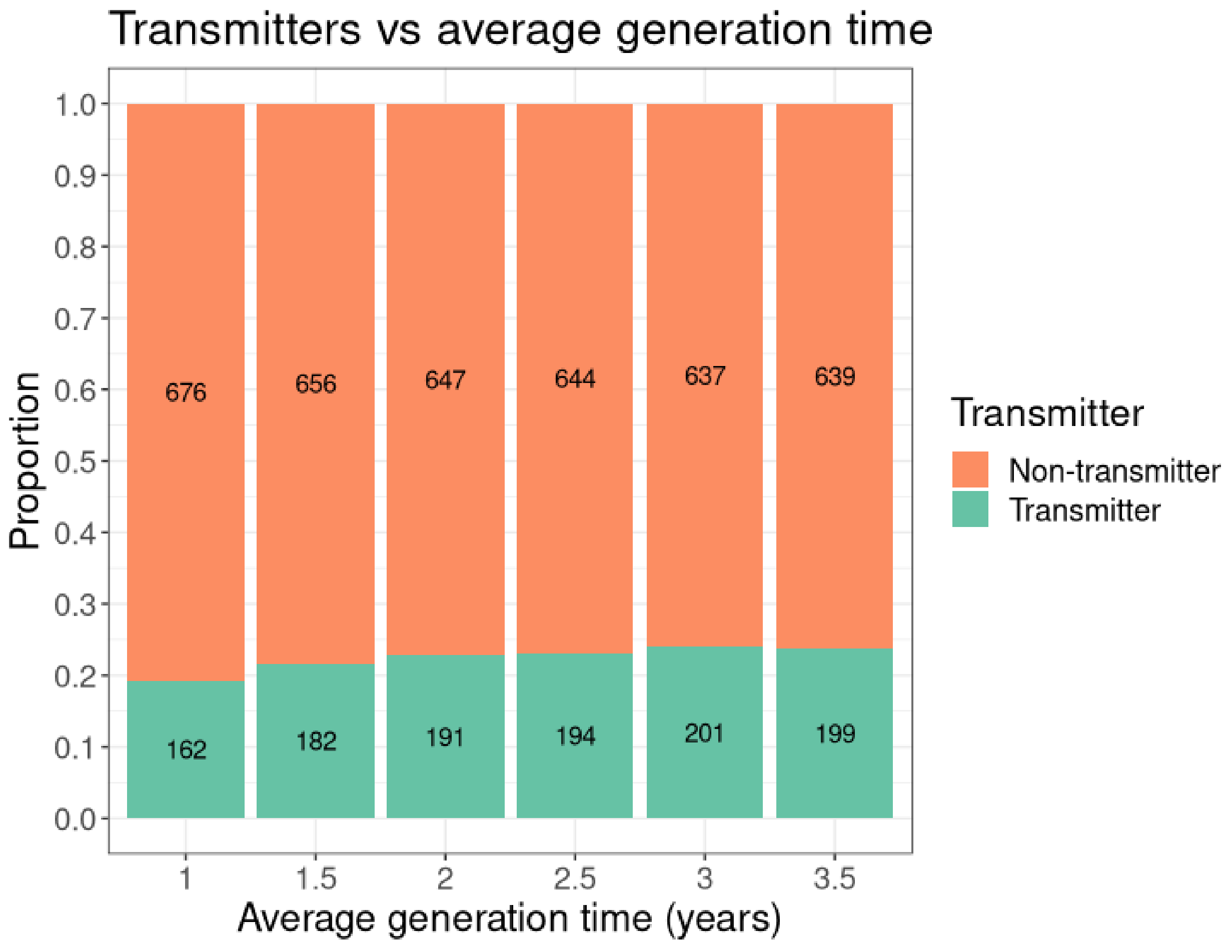

Supplementary Figure 5: Results of the sensitivity analysis. The figure shows the number and proportion of transmitters inferred by TransPhylo when using different priors for the gamma distributions that describe the generation and sampling times (scale 1 and shapes 1, 1.5, 2, 2.5, 3 and 3.5). The results of the manuscript refer to the analysis performed with scale 1 and shape 1.5.

Bibliography

1 Goig GA, Blanco S, Garcia-Basteiro AL, Comas I. Contaminant DNA in bacterial sequencing experiments is a major source of false genetic variability. *BMC Biol* 2020; **18**: 24.

2 Bolger AM, Lohse M, Usadel B. Trimmomatic: a flexible trimmer for Illumina sequence data. *Bioinformatics* 2014; **30**: 2114–20.

3 GitHub - jstjohn/SeqPrep: Tool for stripping adaptors and/or merging paired reads with overlap into single reads. GitHub. https://github.com/jstjohn/SeqPrep (accessed Oct 17, 2022).

4 Li H, Durbin R. Fast and accurate short read alignment with Burrows-Wheeler transform. *Bioinformatics* 2009; **25**: 1754–60.

5 GitHub - broadinstitute/picard: A set of command line tools (in Java) for manipulating high-throughput sequencing (HTS) data and formats such as SAM/BAM/CRAM and VCF. GitHub. https://github.com/broadinstitute/picard (accessed Oct 17, 2022).

6 McKenna A, Hanna M, Banks E, *et al.* The Genome Analysis Toolkit: a MapReduce framework for analyzing next-generation DNA sequencing data. *Genome Res* 2010; **20**: 1297–303.

7 Li H, Handsaker B, Wysoker A, *et al.* The Sequence Alignment/Map format and SAMtools. *Bioinformatics* 2009; **25**: 2078–9.

8 Koboldt DC, Zhang Q, Larson DE, *et al.* VarScan 2: somatic mutation and copy number alteration discovery in cancer by exome sequencing. *Genome Res* 2012; **22**: 568–76.

9 Cingolani P, Platts A, Wang LL, *et al.* A program for annotating and predicting the effects of single nucleotide polymorphisms, SnpEff: SNPs in the genome of Drosophila melanogaster strain w1118; iso-2; iso-3. *Fly* 2012; **6**: 80–92.

10 Menardo F, Duchêne S, Brites D, Gagneux S. The molecular clock of *Mycobacterium tuberculosis*. *PLoS Pathog* 2019; **15**: e1008067.

11 Minh BQ, Schmidt HA, Chernomor O, *et al.* IQ-TREE 2: New Models and Efficient Methods for Phylogenetic Inference in the Genomic Era. *Mol Biol Evol* 2020; **37**: 1530–4.

12 To T-H, Jung M, Lycett S, Gascuel O. Fast Dating Using Least-Squares Criteria and Algorithms. *Syst Biol* 2016; **65**: 82–97.

13 Menardo F, Duchêne S, Brites D, Gagneux S. The molecular clock of *Mycobacterium tuberculosis*. *PLoS Pathog* 2019; **15**: e1008067.

14 Menardo F. Understanding drivers of phylogenetic clustering and terminal branch lengths distribution in epidemics of. *Elife* 2022; **11**. DOI:10.7554/eLife.76780.

15 Didelot X, Gardy J, Colijn C. Bayesian inference of infectious disease transmission from whole-genome sequence data. *Mol Biol Evol* 2014; **31**: 1869–79.
